# Identification of QTLs for powdery mildew (*Podosphaera aphanis;* syn. *Sphaerotheca macularis* f. sp. *fragariae*) susceptibility in cultivated strawberry (*Fragaria ×ananassa*)

**DOI:** 10.1371/journal.pone.0222829

**Published:** 2019-09-19

**Authors:** Daniel J. Sargent, Matteo Buti, Nada Šurbanovski, May Bente Brurberg, Muath Alsheikh, Matthew P. Kent, Jahn Davik

**Affiliations:** 1 PlantSci Consulting Ltd. Kent, United Kingdom; 2 Fondazione Edmund Mach, San Michele all’Adige, Trentino, Italy; 3 Department of Agriculture, Food, Environment and Forestry, University of Florence, Florence, Italy; 4 Division of Biotechnology and Plant Health, Norwegian Institute of Bioeconomy Research, Ås, Norway; 5 Department of Plant Sciences, Norwegian University of Life Sciences, Ås, Norway; 6 Graminor Breeding Ltd., Ridabu, Norway; 7 Department of Animal and Aquacultural Sciences, Norwegian University of Life Sciences, Ås, Norway; USDA-ARS Southern Regional Research Center, UNITED STATES

## Abstract

Strawberry powdery mildew (*Podosphaera aphanis* Wallr.) is a pathogen which infects the leaves, fruit, stolon and flowers of the cultivated strawberry (*Fragaria ×ananassa*), causing major yield losses, primarily through unmarketable fruit. The primary commercial control of the disease is the application of fungicidal sprays. However, as the use of key active ingredients of commercial fungicides is becoming increasingly restricted, interest in developing novel strawberry cultivars exhibiting resistance to the pathogen is growing rapidly. In this study, a mapping population derived from a cross between two commercial strawberry cultivars (‘Sonata’ and ‘Babette’) was genotyped with single nucleotide polymorphism (SNP) markers from the Axiom iStraw90k genotyping array and phenotyped for powdery mildew susceptibility in both glasshouse and field environments. Three distinct, significant QTLs for powdery mildew resistance were identified across the two experiments. Through comparison with previous studies and scrutiny of the *F*. *vesca* genome sequence, candidate genes underlying the genetic control of this trait were identified.

## Introduction

Strawberry powdery mildew (*Podosphaera aphanis* Wallr., previously known as *Sphaerotheca macularis* f.sp. *fragariae*) infects the leaves, fruit, stolon and flowers of strawberry plants and causes major yield losses where the infection renders the fruit unmarketable. Many commercial strawberry varieties exhibit high levels of susceptibility to the disease [1] and while brief exposure to UV-C radiation has been reported to effectively reduce the mildew symptoms on strawberry [2], commercial control of the disease is primarily through the application of fungicidal sprays. However, since new European regulations are restricting the use of active ingredients of commercial fungicides [3] and the efficacy of triazole fungicides as a powdery mildew control in strawberry is reducing [4], alternative disease control methods are now required for sustainable strawberry cultivation, particularly under protected canopies[5].

The potential of breeding for powdery mildew resistance in strawberry has been explored previously [1,6]. In the study of Davik and Honne [6], both broad- and narrow-sense heritability for resistance to powdery mildew was found to be intermediate, and a number of commercial cultivars, such as ‘Solprins’, ‘Kent’ and ‘Patty’, were shown to transmit high levels of resistance to powdery mildew to their progeny when used as parents. More recently, Kennedy et al [7] identified wild octoploid strawberry germplasm with enhanced levels of resistance to powdery mildew, and the authors suggested introgression of such wild material into the cultivated strawberry could be a strategy of novel resistance for breeding future commercial varieties. However, such an approach, particularly if resistance is polygenic, would be challenging.

Powdery mildew species infect many different plant species, and members of the genus *Podosphaera* (previously known as *Sphaerotheca*) are economically significant pathogens of many commercial species of the Rosaceae family (of which strawberry is a member), including apple, apricot, and peach [8]. In cultivated apple (*Malus pumila*), recessive mutations in homologues of *Mildew Resistance Locus O* (MLO; [9]) confer stable resistance to powdery mildew, often considered as immunity to the disease [8]. Indeed, Pessina et al [10] reported that knock-down expression of the *MLO* gene *MdMLO19* in apple reduced the susceptibility of transformed lines to powdery mildew, and Jiwan et al [11] reported that antisense expression of the peach *MLO* gene *PpMLO1* in transgenic cultivated strawberry lines conferred resistance to *Fragaria*-specific powdery mildew isolates. Recently, two groups [12,13] characterised several *MLO* genes and pseudogenes in the genome sequence of *F*. *vesca*. Twelve of the identified genes were expressed following challenge by isolates of powdery mildew in *F*. *vesca* accessions [13]. Two of these genes, annotated as FvH4_7g08130.1 and FvH4_7g19790.1 in the FvH4 v4.0 assembly [14], were differentially expressed in response to powdery mildew infection in *F*. *vesca* [13].

Markers associated with strawberry powdery mildew resistance were described in a US patent application by Koishihara et al [15], who reported the identification of a major resistance locus in segregating populations derived from the cultivar ‘Miyazaki Natsu Haruka’ and the breeder’s selection ‘08 To-f’. The locus was located on the proximal end of an unnamed linkage group in the patent, but the authors presented sequence information defining the region from which the QTL was derived. More recently, Cockerton et al [16], using two strawberry mapping populations, reported both stable and transient QTLs for powdery mildew resistance of moderate effect distributed across the cultivated strawberry genome. Using data generated with the strawberry Axiom arrays [17,18], Cockerton et al [16] identified six loci exhibiting stable resistance between populations and years, along with multiple other QTLs that were identified on only one occasion. The authors suggested that the use of the markers defining the QTL in a program of marker-assisted breeding or genomic selection would enable the development of cultivars with increased resistance to powdery mildew. Nelson et al. [1] studied the inheritance of resistance to mildew in strawberry in both glasshouse and field environments. Whilst they concluded that genetic differences between cultivars were expressed similarly in both environments, only moderate, and only occasionally significant correlations were observed between glasshouse and field phenotypic rankings when disease pressure was not extremely high, and weak correspondence between genetic resistance mechanisms at differing severities of infection, as often occur in different environments, were observed.

In this study, a previously published mapping population derived from a cross between the commercial cultivars ‘Sonata’ and ‘Babette’ [19] was supplemented with markers from the Axiom iStraw90k genotyping array [17]. Since previous studies have shown disease pressure to be very different for fungal pathogens in different strawberry production environments [5], we speculated that different mechanisms of resistance might operate in these different environments as has been demonstrated previously for powdery mildew [15], and as such the mapping progeny was phenotyped for powdery mildew in both a glasshouse and field environment. The aim of the study was to identify genetic loci controlling resistance to powdery mildew in cultivated strawberry, and by determining the physical position of the QTL identified through the physical positions of flanking genetic markers on the diploid *Fragaria* genome sequence, to identify potential candidate genes conferring resistance.

## Materials and methods

### Plant material development and growing conditions

An experimental population of 145 F_1_ hybrid seedlings was developed from the cross between the Dutch cultivar ‘Sonata’ (♀, ‘Elsanta’ × ‘Polka’) and the Norwegian cultivar ‘Babette’ (♂, ‘Patty’ × ‘Jonsok’) with the aim of studying the genetics of resistance to powdery mildew in the cultivated strawberry. In previous field trials at the Norwegian Institute of Bioeconomy Research the parental cultivars had shown different responses to powdery mildew, with ‘Sonata’ displaying more robust resistance.

A linkage map of the ‘Sonata’ × ‘Babette’ F_1_ population developed using double digest restriction-site associated DNA sequencing (ddRAD) markers was previously reported by Davik et al [19]. The F_1_ progeny were grown in 1 litre pots and, from each plant, an abundant number of runner plants were cut and raised in 66 pot plastic trays placed in a heated glasshouse under 18-hour days at 20 ± 2°C and 6 hour nights at 14 ± 2°C. Supplemental light was provided by high-pressure sodium lamps (SON-T) at a PPFD of 90 μmol quanta m^-2^ s^-1^. Equal sized plantlets were selected as plant material for one field experiment and one glasshouse experiment. During plant multiplication from runners, powdery mildew control was undertaken using sulphur vapor.

### Field experiment

A duplicated experiment with 137 ‘Sonata’ × ‘Babette’ F_1_ progeny, and additional commercial cultivars was established with an α-design [20] at the experimental site of Graminor Ltd in Ridabu, Norway in August 2013. Additional commercial cultivars, namely the two parental lines, ‘Sonata’ and ‘Babette’, the grandparents, ‘Elsanta’, ‘Polka’, ‘Patty’ and ‘Jonsok’, and the great grandparents, ‘Gorella’, ‘Holiday’, ‘Sivetta’, ‘Honeoye’, ‘Marmolada’ and ‘Senga Sengana’ were included in the trial. Each plot consisted of six clonally replicated plants, and thus in the trial, each experimental unit, i.e., a F_1_ seedling or a cultivar, was represented by 12 plants. The field was allowed to establish before it was covered with two layers of fleece to protect against low-temperature damage during winter. Standard growing regimes were used except for the omission of powdery mildew fungicide treatment. A growing system with plastic mulch and a nutrient solution containing 7.8 mmol N, 1 mmol P, and 4.6 mmol K per litre, applied in a 1:100 ratio to equal approximately the recommended 60 kg N ha^-1^ over the entire growing season was used.

### Glasshouse experiment

The experiment was set up in triplicate with 145 F_1_ progeny and the same commercial cultivars as in the field experiment giving an experiment of 216 plots per replication (glasshouse compartment) using an α-design [20]. The F_1_ progeny were usually present in all three replicates, while the commercial cultivars were represented at varying levels.

Equal sized plants were selected and grown in 9 cm pots using 18 pot trays (Vefi System tray PR909) containing a peat-based potting compost (90% peat, 10% clay), with the addition of 1:5 (v/v) granulated perlite. The plants were raised in a heated glasshouse in the same manner as stated previously. The same nutrient solution used in the field experiment was applied when necessary, usually every second day. One experimental plot consisted of four clonal plants planted in one half of a tray with 18 (3×6 9 cm pots, Vefi System tray PR909). Each tray carried two four-plant plots (S1 Fig), i.e., each F_1_ progeny was represented by 12 clonally propagated plants and the entire F_1_ population by 1,740 plants. Between the experimental plants, one previously infected plant of the susceptible cultivar ‘Korona’ was included.

### Inoculum preparation

An isolate of strawberry powdery mildew was collected from infected ‘Korona’ leaves from glasshouse grown plants and multiplied on surface sterilized young unfolded leaves of susceptible ‘Korona’ placed in dishes with water agar [21]. A batch of uninfected ‘Korona’ plantlets were inoculated by gently rubbing their leaves against the infected leaves and the pathogen was allowed to establish for three weeks before putting one plant in the middle of each plot (S1 Fig). On each corner of the three replicates, fans were placed and run for one hour in the morning and one hour in the afternoon to distribute spores from the infected source plants.

### Disease scoring

Individual plants of the experiments were phenotyped using the five point scale described by Simpson [22]:

1. No visual symptoms
2. Slight leaf curling, no apparent mycelia
3. Leaf curling and mottling
4. Severe leaf curling, reddening and visible damage to lower leaf surface;
5. Severe necrosis and some leaf death.

The scores were made by a single qualified person three times every second week starting on October 7^th^ 2013 in the glasshouse experiment, and every second week from early June to the end of the fruiting season in the field experiment. Using the trapezoidal formula, the area under the curve score was calculated for each genotype, along with mean and end-point scores for each genotype.

### Spatial modelling

Even though means were taken to ensure an even spread of inoculum throughout the experiments, it is conceivable that this not always happened. Such unevenness can be one cause for correlations between neighbouring plots. In order to account for this, we used the approach taken by Gilmour et al [23], where correlations between neighbouring plots and trends (linear and cubic) across the experimental fields can be accounted for. When modelling these experimental data, we followed the sequential approach taken by Davik and Honne [6]:

1. Initially a base model was established, typical for a replicated experiment with independent errors
2. Then the dependency of adjacent plots was modelled through the error matrix (R) using a two-dimensional autoregressive spatial model, often referred to as AR1 × AR1
3. Finally, other fixed or random design or trend effects were added.

The significance of the AR1 × AR1 spatial error model and the random effects were evaluated using the likelihood ratio [24]. The fixed effects were evaluated partly by the accompanying F-statistics and partly inspecting a variogram of the residuals. The statistical analyses of the field experiments were done using the ASReml software [25].

### Linkage map supplementation

The linkage map of Davik et al [19] developed using ddRAD markers was supplemented with SNP markers from the Axiom iStraw 90k array [17] that segregated in the progeny. DNA from the progeny was extracted using the DNeasy Plant Minikit (Qiagen) and assessed for quality using a Qiagility spectrophotometer (Qiagen). Samples with a 260/280 ratio of between 1.8 and 2.0 were normalised to 10 ng/μl following quantification on a Qubit fluorometer against manufacturer-supplied standards (Thermo Scientific). DNA from the parental genotypes ‘Sonata’ and ‘Babette’ and all progeny from the initial population of 145 seedlings for which phenotypic data were available was genotyped on a GeneTitan instrument (ThermoFisher) using the Axiom 96-array protocol. SNP genotypes were assigned according to the best-practices protocol using the Axiom Analysis Suite software (ThermoFisher) and in total 113 seedlings had both a genotype and phenotype. SNP data from the parental genotypes were examined, and those SNPs that were heterozygous in at least one parent were retained, whilst all SNPs that were either monomorphic or where there were missing data for either parental genotype were discarded. The SNP segregation data were retained for further analysis if the progeny contained only genotypes predicted from the parental combination and contained 8% or fewer missing values.

Segregating SNPs were coded for analysis with JOINMAP 4.1 (Kyazma, NL) and data were combined with the segregation data used to construct the previously published map of the ‘Sonata’ × ‘Babette’ mapping population [19]. Marker groups were determined and retained based on the previously published linkage groups, and marker inclusion and ordering within a linkage group was based on the criteria used for the construction of the previously published ddRAD linkage map [19]; marker placement was determined using regression mapping with a minimum logarithm of odds (LOD) score threshold of 3.0, a recombination fraction threshold of 0.35, ripple value of 1.0, jump threshold of 3.0 and a triplet threshold of 5.0, and mapping distances were calculated using the Kosambi mapping function to produce a linkage map. Linkage group names and sub-genomes (A, b, X1 and X2) were assigned according to the presence of Axiom SNP markers common to the map of Sargent et al [26] and the linkage maps presented were plotted using MapChart 2.1. BLAST analysis of the *F*. *vesca* ‘Hawaii 4’ v4.0 genome sequence (FvH4 v4.0) and the *F*. *×ananassa* ‘Camarosa’ v1.0 genome sequence [27] was performed with default parameters using the ddRAD tag sequences and the published Axiom SNP flanking sequences as queries [14,28] and the physical positions of the markers were used to plot Marey maps of genetic vs. physical position of all mapped genetic markers.

### Quantitative trait loci detection

End-point (EP) and mean (MP) susceptibility scores, and area under disease progress curve (AUDPC), for both the field and glasshouse evaluation of mildew susceptibility were used to perform QTL analyses on the 113 progeny for which phenotypic data from both field and glasshouse were available and that had both a ddRAD and Axiom genotype set. Data were analysed by interval mapping and the phenotypic variance explained by each marker genotype and associated LOD values were calculated using MapQTL 6.0 (Kyazma, NL). A genome-wide LOD threshold of 4.5 was determined following a permutation test over 20,000 permutations for end-point, mean and AUDPC datasets. The restricted multiple QTL method (rMQM) was performed using markers with significant association with mildew susceptibility as cofactors to attempt uncover the presence of minor QTLs throughout the linkage groups of the genetic map. The step size used for both the interval mapping (IM) and the restricted multiple QTL mapping (rMQM) was 1.0 cM.

### Candidate gene identification and annotation

Genes located in the 3 Mb region of the FvH4 v4.0 genome sequence centred around the marker most significantly associated with each QTL were scrutinized to identify candidate genes putatively involved in powdery mildew resistance. Using the best-hit reports of homology of predicted transcripts within the QTL regions, determined using the blastx algorithm against the NCBI non-redundant (Release 2017–07), *Arabidopsis* (TAIR10), UniProt SwissProt (Release 2017–11), and UniProt TrEMBL (Release 2017–11) protein databases that were downloaded from the Genome Database for Rosaceae (GDR) [29], along with the gene model annotation on the GDR, candidate genes with a putative role in disease resistance were identified.

## Results

### Powdery mildew resistance phenotyping

**Fig 1:**
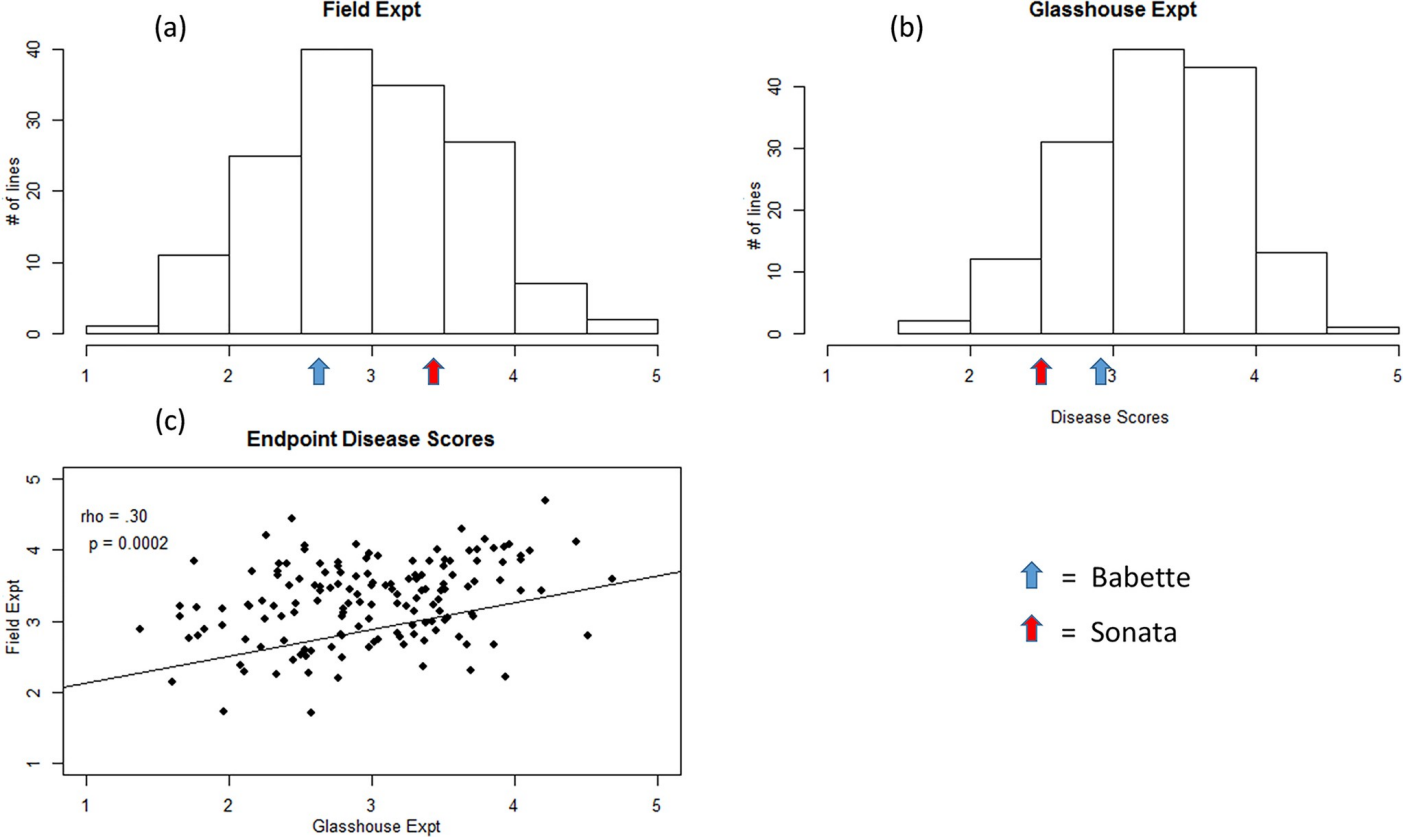
The average end-point mildew susceptibility phenotypic scores of the ‘Sonata’ × ‘Babette’ F_1_ mapping progeny for the field experiment (a) and glasshouse experiment (b), along with the Spearman’s rank correlation between the two datasets (c).

The average end-point score in the field experiment was 2.9 with a minimum and maximum score of 1.4 and 4.7 (Fig 1A). For the glasshouse experiment the mean score was 3.3 and the minimum and maximum scores were 1.7 and 4.7 (Fig 1B). The results showed that in under field conditions, ‘Babette’ was more resistant to powdery mildew, whilst under glasshouse conditions, both parental cultivars showed similar levels of resistance to the pathogen, with ‘Sonata’ being slightly more resistant than ‘Babette’. Within both experiments the differences between the 145 entries were highly significant (p < 0.0001, Wald F test) indicating a strong genetic regulation of powdery mildew resistance/susceptibility in both environments. Even though the Spearman’s rank correlations between the two environments was highly significant, the correlation itself was not high, indicating rank shifts from the field experiment to the glasshouse experiment (ρ = 0.30, p = 0.0002) (Fig 1C).

### Linkage map supplementation

**Fig 2:**
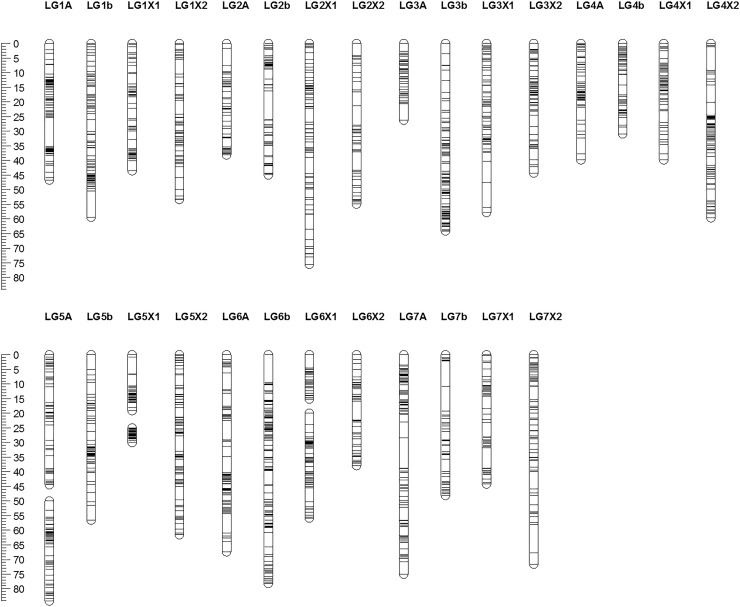
The ‘Sonata’ and ‘Babette’ F_1_ linkage map containing 3,550 markers at 1,474 loci in 31 linkage groups representing the 28 haploid chromosomes of the *F*. *×ananassa* genome. The linkage map spans a total genetic distance of 1498.2 cM and a total physical distance of 769.9 Mbp, estimated from the physical positions of the distal- and proximal-most markers on the diploid *F*. *vesca* v4.0 genome sequence. All linkage groups have been assigned to one of the four *F*. *×ananassa* homeologous subgenomes (i.e. A, b, X1 or X2) of the linkage map of Sargent et al [26] through identification of common markers in the two studies.

**Fig 3:**
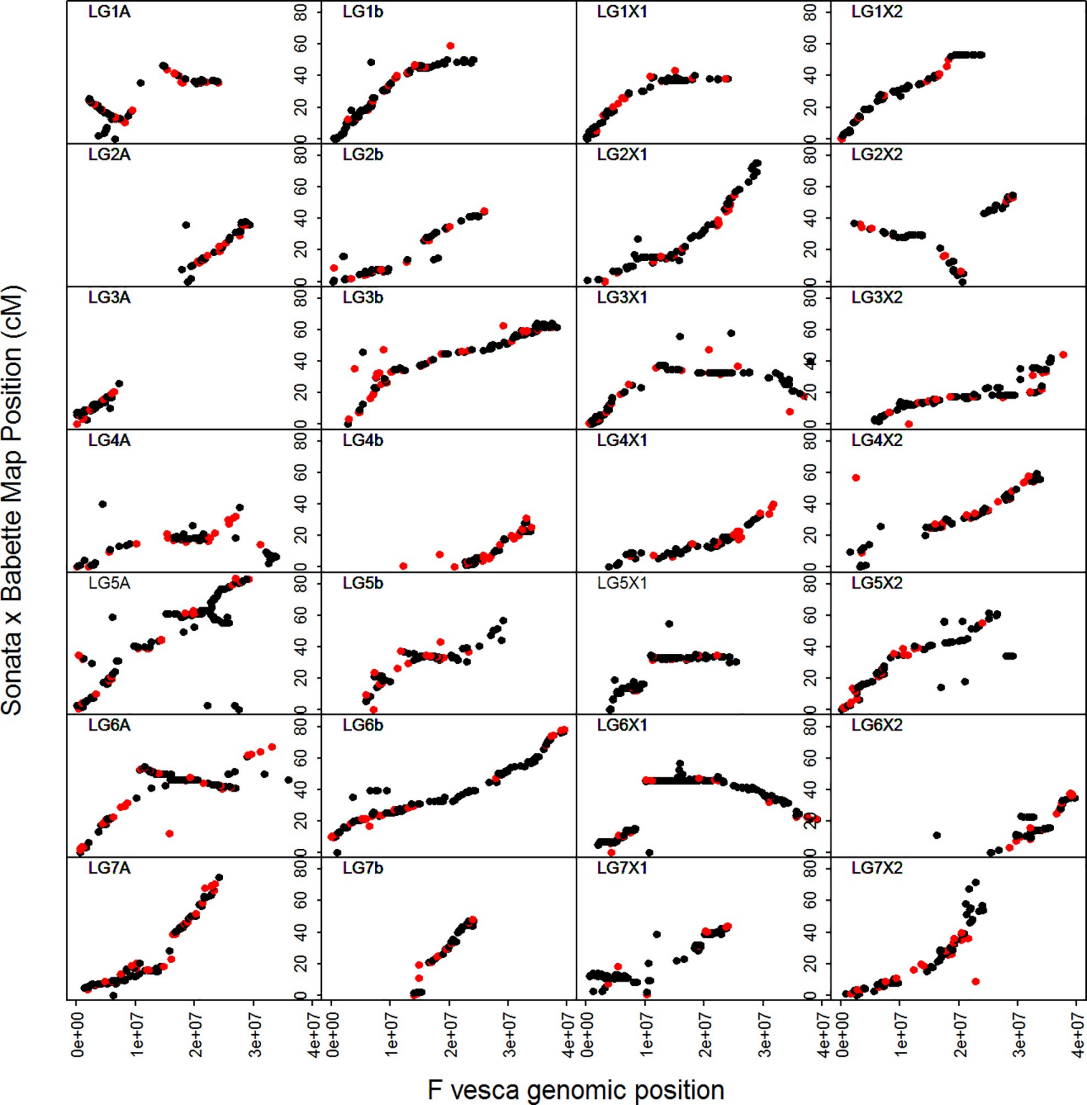
Marey maps depicting the relationships and colinearity between the markers’ physical position on the *F*. *vesca* v4.0 genome sequence and the genetic positions on the ‘Sonata’ and ‘Babette’ linkage groups. Genetic distances are plotted on the y-axes and physical distances on the x-axes. Red points indicate the positions of ddRAD markers, whilst black points indicate the positions of Axiom iStraw90 markers.

The ‘Sonata’ × ‘Babette’ linkage map was constructed using data from a total of 113 progeny for which both phenotypic and genotypic data were available (S1 File). A total of 580 ddRAD markers from the linkage map of Davik et al [19] were assimilated with 2,970 markers from the Axiom iStraw 90k array at 1,474 loci into 31 linkage groups, representing the 28 haploid chromosomes of the *F*. *×ananassa* genome (Fig 2; Table 1). The markers spanned a total genetic distance of 1498.2 cM, and a total physical distance of 769.9 Mbp, estimated from the physical positions of the distal- and proximal-most markers on the diploid FvH4 v4.0 genome sequence [28,30]. Identification of common markers with the linkage map of Sargent et al [26] enabled all 31 linkage groups to be assigned to one of the four *F*. *×ananassa* homeologous subgenomes (i.e. A, b, X1 or X2 according to the nomenclature of Sargent et al [26]) (Fig 2). Marey maps depicting the genetic distances of the linkage groups resolved vs the physical positions of the markers on the FvH4 v4.0 genome sequence [14] showed overall good marker collinearity between the genetic and physical maps (Fig 3).

**Table 1:** Summary of the molecular markers mapped to the 31 linkage groups of the ‘Sonata’ × ‘Babette’ F_1_ mapping population, detailing the number of ddRAD and Axiom markers mapped to each linkage group, the linkage group lengths and the physical distances on the *F*. *vesca* genome associated with each linkage group.

Linkage group	ddRAD	Axiom	Total	LG length (cM)	LG physical length (Kbp)
LG1A	32	92	124	46.7	23,957,603
LG1b	19	147	166	59.4	23,685,054
LG1X1	16	68	84	43.5	24,060,629
LG1X2	11	98	109	53.3	23,779,102
LG2A	17	48	65	38.1	11,484,408
LG2b	13	63	76	44.9	25,698,315
LG2X1	20	86	106	75.5	28,877,060
LG2X2	18	59	77	54.9	30,310,907
LG3A	16	106	122	26.2	7,150,320
LG3b	36	107	143	64.1	37,489,006
LG3X1	19	113	132	57.8	37,494,884
LG3X2	17	134	151	44.3	31,899,046
LG4A	20	75	95	39.8	33,800,549
LG4b	26	48	74	30.9	21,646,936
LG4X1	16	80	96	39.8	31,159,059
LG4X2	22	70	92	59.6	32,958,309
LG5A.1	21	91	112	44.4	27,514,567
LG5A.2	12	153	165	34.2	23,721,032
LG5b	18	88	106	56.5	23,221,682
LG5X1.1	4	63	67	19.1	5,731,902
LG5X1.2	9	127	136	24.8	20,086,173
LG5X2	21	91	112	61.5	29,120,806
LG6A	40	132	172	67.4	35,379,216
LG6b	26	134	160	78.2	39,485,620
LG6X1	19	259	278	56.5	37,111,072
LG6X2	15	78	93	37.9	23,459,542
LG7A	32	150	182	75	22,775,856
LG7b	14	41	55	48.1	10,165,563
LG7X1	12	119	131	44.2	23,438,682
LG7X2	19	50	69	71.6	23,258,857
Total	580	2970	3550	1498.2	769,921,757

### QTL analysis and detection

**Fig 4:**
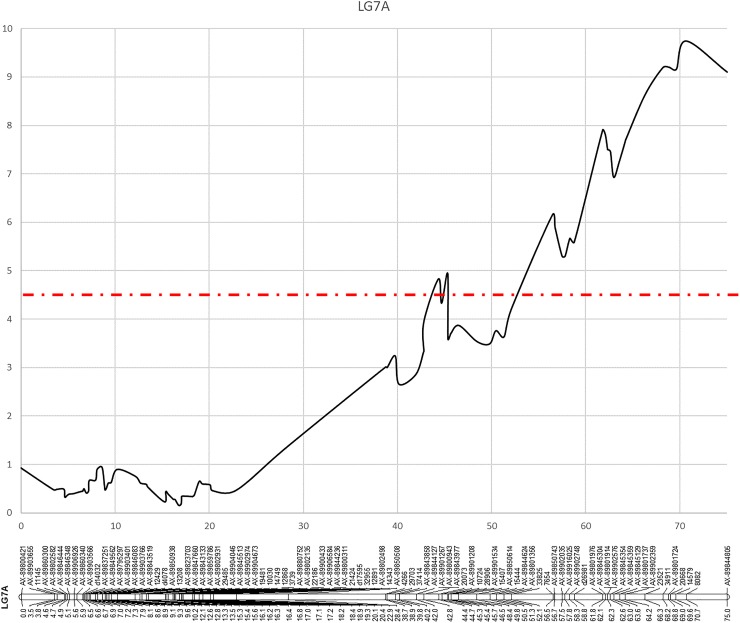
Plots of the significant QTLs (LOD threshold >4.5) identified for mildew susceptibility on linkage group LG7A in the glasshouse mildew evaluation of the ‘Sonata’ × ‘Babette’ mapping population. LOD scores are plotted along the x-axes (dotted line indicates the 4.5 LOD threshold), whilst genetic distances are plotted along the y-axes.

**Fig 5:**
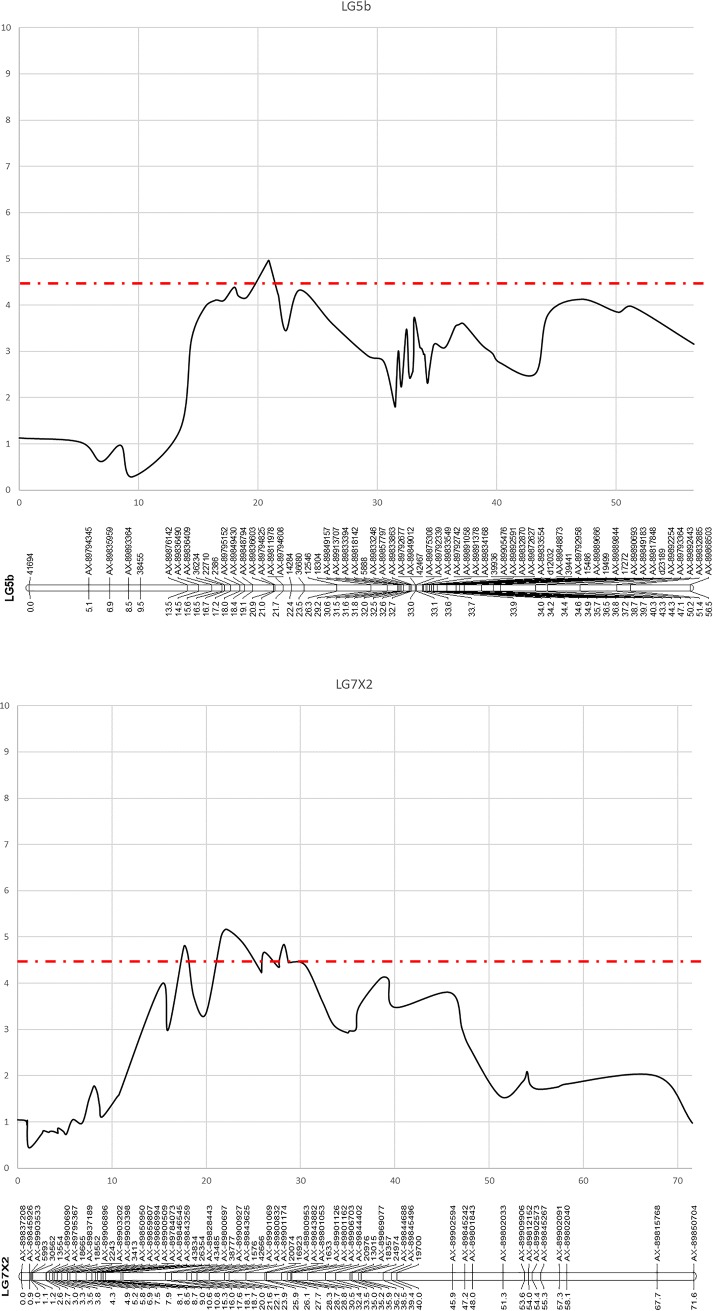
Plots of the significant QTLs (LOD threshold >4.5) identified for mildew susceptibility on linkage group LG7A in the glasshouse mildew evaluation in the field evaluation of the ‘Sonata’ × ‘Babette’ mapping population. LOD scores are plotted along the x-axes (dotted line indicates the 4.5 LOD threshold), whilst genetic distances are plotted along the y-axes.

The AUDPC, along with the end-point (EP) and mean (MP) disease scores were used for QTL analysis using the interval mapping approach and the non-parametric rank sum test of Kruskal-Wallis. In all analyses, three significant QTLs were identified; two in the field experiment and one in the glasshouse experiment (S1 File). Details of the positions of the QTLs identified for each of the datasets following interval mapping, including linkage group, genetic and physical positions of the closest flanking markers, and associated LOD values and total phenotypic variance explained, are given in Table 2, along with the associated Kruskall-Wallis test statistic (K*) scores for the most significant marker in each dataset. A significant QTL above the LOD threshold of 4.5 was identified for mildew resistance on linkage group LG7A of the ‘Sonata’ × ‘Babette’ linkage map in the 2013 glasshouse mildew evaluation (Fig 4) with resistance alleles segregating in the ‘Babette’ parental genotype, whilst two significant QTLs above the LOD threshold of 4.5 were identified on linkage groups LG5b and LG7X2 in the 2015 field evaluation (Fig 5), with resistance alleles segregating in the ‘Babette’ and in both parental genotypes on LG5b and LG7X2 respectively. In both the glasshouse and field evaluations, the LOD peaks of the identified QTLs were higher in the end-point data analysis. The QTLs were associated with ddRAD marker 8082 (LOD = 9.74, percentage variance explained = 34.2%, equating to a one point change on the disease ranking scale used to phenotype the plants) on LG7A in the glasshouse evaluation, and with Axiom markers AX-89836603 (LOD = 4.97, percentage variance explained 18.3%, equating to a change of approximately 0.5 on the disease ranking scale used to phenotype the plants) and AX-89800832 (LOD = 5.17, percentage variance explained 19%, equating to a change of approximately 0.5 on the disease ranking scale used to phenotype the plants) on LG5b and LG7X2, respectively. BLAST analysis of the sequence data available for the most significant mapped markers against the FvH4 v4.0 genome sequence (S1 File) placed marker 8082 (LG7A) on chromosome 7 at a physical position of 23,480,779 bp. Marker AX-89836603 (LG5b) was located on chromosome 5 at a physical distance of 7,298,636 bp, whilst marker AX-89800832 (LG7X2) was located on chromosome 7 at a physical distance of 16,764,242 bp. The positions of the markers were also queried on the *F*. *×ananassa* ‘Camarosa’ genome sequence and whilst the positions were largely concordant with those of the FvH4 v4.0, the individual ‘Camarosa’ homeologues of both Fvb5 and Fvb7 within the genome were orientated in opposite directions, making positioning of markers between homeologues confusing, and identification of candidate gene positions problematic. The positions of mapped markers and subsequent identification of candidate genes was therefore performed using the FvH4 v4.0 diploid genome sequence.

**Table 2:** The positions of the significant QTLs (LOD >4.5) identified for AUDPC, endpoint and mean disease score datasets following interval mapping, including linkage group, genetic and physical positions of the closest flanking markers, and associated LOD values and total phenotypic variance explained, along with the associated Kruskall-Wallis test statistic (K*) scores for the most significant marker in each dataset.

QTL name	Experiment	Linkage group	Closest SNP	Genetic position	Physical position FvH4.4	LOD	Percentage variance explained	1-LOD interval
FxaPMR7A	Glasshouse	LG7A	8082	70.7 cM	23,480,779 bp	9.74	34.2%	66.3–75.0 cM
FxaPMR5b	Field	LG5b	AX-89836603	20.9 cM	7,298,636 bp	4.97	18.3%	15.6–23.5 cM
FxaPMR7X2	Field	LG7X2	AX-89800832	22.2 cM	16,764,242 bp	5.17	19%	19.9–30.5 cM

### Candidate gene identification

**Fig 6:**
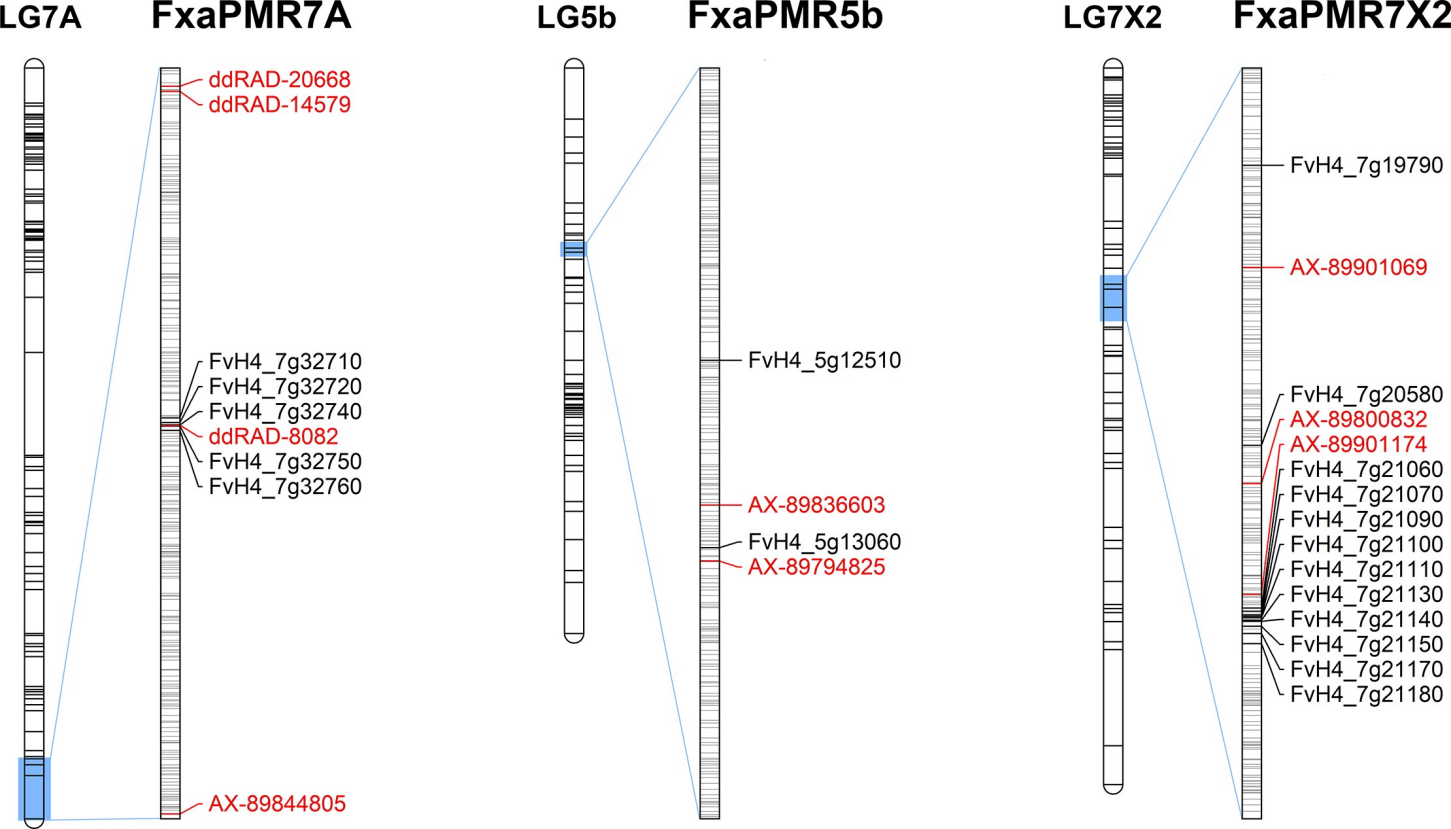
The physical positions of the candidate resistance genes associated with powdery mildew resistance in the ‘Sonata’ × ‘Babette’ mapping population. The three genetic linkage groups on which resistance QTL were identified; LG7A in the 2013 glasshouse experiment and LG5b and LG7X2 in the 2015 field trials are shown, along with local physical maps of the QTL regions showing flanking genetic markers in red, candidate resistance genes identified in blue, and intervening non-candidate genes in grey. Genetic distances are represented in centiMorgans (cM) whilst physical distances are represented in nucleotides.

Analysis of gene predictions in the physical regions surrounding the QTLs using tblastx revealed several candidate resistance genes. In the QTL interval on LG7A, clusters of candidate resistance genes of the TIR-NBS-LRR class were identified. Five genes (FvH4_7g32710, 7g32720, 7g32740, 7g32750 and 7g32760) were clustered in an interval between 23,469,310 and 23,493,387 bp containing the 8082 ddRAD SNP marker most significantly associated with the LG7A QTL, the closest being FvH4_7g32740, positioned at 23,480,734, just 45 bp from marker 8082. In the QTL interval on LG7X2, FvH4_7g20580, a predicted NB-ARC domain-containing disease resistance gene was identified at 16,702,990 bp, 61 kb from AX-89800832, the Axiom marker most significantly associated with the LG7X2 QTL. The *MLO* gene *FvMLO-20* (FvH4_7g19790) was positioned 0.5 Mb from AX-89800832, and a cluster of ten TIR-NBS-LLR resistance genes (FvH4_7g21060, 7g21070, 7g21090, 7g21100, 7g21110, 7g21130, 7g21140, 7g21150, 7g21170, and 7g21180) were positioned in an interval between 16,963,133 and 17,059,456, i.e. 199 kb from AX-89800832. On chromosome 5, two candidate resistance genes were identified in the 100 kb surrounding marker AX-89836603 in the QTL interval; gene FvH4_5g12510, a predicted leucine-rich receptor-like protein kinase, positioned at 7,370,161 bp, and gene FvH4_5g13060, a predicted serine/threonine protein kinase 3, located at 7,366,880 bp, the closest of which was 68,244 bp from marker AX-89836603. Fig 6 shows the three linkage groups on which the QTL for powdery mildew resistance were identified, along with the physical positions of the closest mapped molecular markers, the candidate resistance genes and the positions of the intervening non-candidate genes.

## Discussion

Previous investigations of mildew resistance in strawberry have identified multiple QTLs exerting small effects controlled by both additive and non-additive genetic components [6,22]. In total, three significant QTLs for powdery mildew resistance were identified in this investigation; a single QTL on linkage group LG7A according to the nomenclature of Sargent et al [26] from a glasshouse-based disease trial, and two QTLs from a field-based experiment. Whilst similar trends were observed in disease progression in both glasshouse and field evaluation of mildew resistance in this investigation, a strong genotype × environment interaction was detected between experiments as with previous studies of the genetics of mildew resistance in strawberry [7,16]. Additionally, quite different QTLs were identified in the field and glasshouse studies.

In the glasshouse experiment, the marker most closely associated with the resistance QTL on the ‘Sonata’ × ‘Babette’ linkage map was the ddRAD marker 8082, physically located at 23,654,621 bp on chr7 of the *F*. *vesca* v4.0 genome sequence, with the 1-LOD markers flanking marker 8082 spanning the physical interval between 23,335,372 and 24,102,125 bp. Koishihara et al [15] filed a patent application characterising a major QTL for powdery mildew resistance in Japanese accessions of the cultivated strawberry. In their application, they detailed DNA sequence for 19 molecular markers distributed within the genomic interval containing their QTL. BLAST analysis of the marker sequences performed in this investigation (S1 File) located the associated markers to an interval of chr7 of the *F*. *vesca* v4.0 genome sequence between 21,061,818 bp and 23,555,440 bp, a very similar physical interval to that of the QTL characterised in this study; the two studies sharing an overlapping region of 220,068 bp which contained the most significantly associated region of the ‘Sonata’ × ‘Babette’ linkage map, including marker 8082. The overlapping physical location of the QTL identified in the glasshouse experiment of this study and the QTL reported by Koishihara et al [15] indicates that they are orthologous QTLs, despite being characterised based on commercial strawberry cultivars from highly divergent genetic backgrounds, under different environmental conditions, and presumably using very different strains of powdery mildew.

In the physical proximity of the QTL, a cluster of five TIR-NBS-LRR resistance genes were identified, the closest being 45 bp from marker 8082. Such genes are members of the largest disease resistance gene family in plants, and their cognate proteins contribute to signal transduction during plant defence [31]. TIR-NBS-LRR genes encode proteins containing an amino-terminal variable domain, a central nucleotide binding site (NBS), a carboxy-terminal leucine rich repeat (LRR) domain and a Toll/Interleukin-1 (TIR) receptor domain [32]. Numerous previous studies have demonstrated the role of NBS-LRR in plant disease resistance to many different viral, bacterial and fungal pathogens [33], including the powdery mildew resistance gene *Mla1* (CC-NBS-LRR) in barley [34], the stripe rust resistance gene *γr10* (CC-NBS-LRR) in wheat [35] and the *Peronospora parasitica* resistance gene *Rpp5* (TIR-NBS-LRR) in Arabidopsis [36]. The close physical proximity to the resistance gene clusters to the QTLs identified on LG5b and LG7A suggest a role for TIR-NBS-LRR resistance genes in powdery mildew resistance in cultivated strawberry, however, further functional studies are required in order to determine the role of these genes in powdery mildew resistance.

Recently Miao et al [12] and Jambagi and Dunwell [13] characterised a number of *MLO* genes and pseudogenes in the *F*. *vesca* genome sequence. Twelve of the genes identified were expressed following challenge by powdery mildew in *F*. *vesca* accessions [13]. Two of the genes, annotated as FvH4_7g08130.1 and FvH4_7g19790.1 in the FvH4 v4.0 assembly which were differentially expressed in response to powdery mildew infection in the study of Jambagi and Dunwell [13], were located on chr7 of the FvH4 v4.0 genome sequence at 8,086,217 bp and 16,255,327 bp respectively. The QTL identified on LG7X2 in the analysis of field data in this experiment was associated most strongly with the Axiom marker AX-89800832 which was located at a physical distance of 16,764,302 bp in the FvH4 v4.0 genome sequence. Gene FvH4_7g19790.1 was positioned within the wide 1-LOD interval of the LG7X2 QTL (14,537,753 bp– 19,214,153 bp) and as such is a possible candidate for the gene underlying the QTL.

The identification of different QTLs in the field and glasshouse experiments conducted here, and associated rank shifts in phenotypes between parents and progeny between the two environments suggests, as with previous studies [16], that resistance to powdery mildew is under the control of different genes in different environments. However, the likely orthology of the QTL identified here with a major QTL identified from the segregating populations from an unrelated Japanese study [15] supports the significance and potential utility of the loci identified in this study. These would make excellent candidates for marker development and marker-assisted selection with the potential to perform consistently across a wide germplasm base, and with different mildew strains. Further work to validate the QTL identified here and develop markers for resistance screening would be required to enable resistant cultivar development.

## Supporting information

S1 Fig Plot layout of the greenhouse experiment. Four experimental plants of each F1 line where used in each plot. The figure shows two lines (‘x’ and ‘y’) and the powdery mildew infected source plant.
(TIF)

S1 File Genetic and phenotypic data. Genotyping and phenotyping data used in the analyses in this study, along with the output from the QTL analyses, sequence tag data for the ddRAD and Axiom markers used in the study and their physical positions on the FvH4 v4.0 genome sequence, the genetic positions of all mapped markers, and the blastx and annotation links for all candidate genes identified in this study.
(XLSX)
