# Supplementary figures and images for: Identification of QTLs for powdery mildew (Podosphaera aphanis; syn. Sphaerotheca macularis f. sp. fragariae) susceptibility in cultivated strawberry (Fragaria ×ananassa)
Source: PLoS One. 2019 Sep 19;14(9):e0222829. doi: 10.1371/journal.pone.0222829 (PMC6752805; doi:10.1371/journal.pone.0222829)

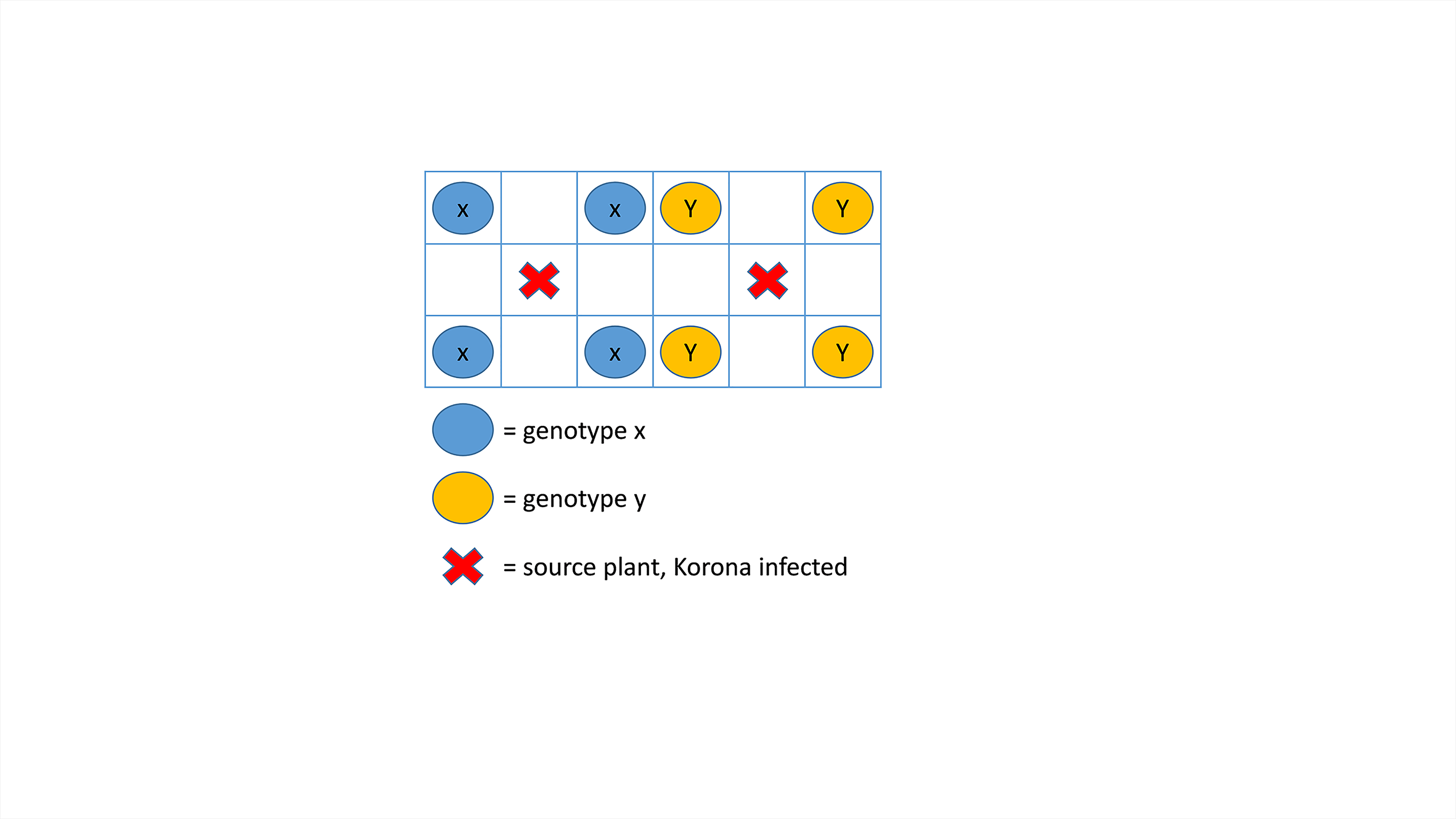

Supplement: S1 Fig — Four experimental plants of each F1 line where used in each plot. The figure shows two lines (‘x’ and ‘y’) and the powdery mildew infected source plant. (TIF) [file pone.0222829.s001.tif]
